# Comparison of Two Different Techniques Of Warfarin Dosing Determination - A Chemometrics Study

**DOI:** 10.22037/ijpr.2019.1100653

**Published:** 2019

**Authors:** Miroslava Makohusová, Viera Mrázová, Adriána Bednárová, Eva Milatová, Jozef Sokol, Marek Pleško, Angelika Bátorová

**Affiliations:** a *Department of Chemistry, Faculty of Natural Sciences, University of SS Cyril and Methodius, Trnava, Slovak Republic. *; b *Department of Pediatric Hematology and Oncology, Comenius University - Faculty of Medicine and National Institute of Children’s Diseases, Bratislava, Slovak Republic *; c *Department of Internal Medicine, Slovak Medical University, University Hospital, Bratislava, Slovak Republic *; d *National Hemophilia Center, Department of Hematology and Transfusion Medicine, Faculty of Medicine of the Comenius University and University Hospital, Bratislava, Slovak Republic.*

**Keywords:** Warfarin dose, Pharmacogenetics, Gene polymorphisms, Passing-Bablock regression, Bland-Altman method

## Abstract

A high prevalence of genetic polymorphisms increases sensitivity to warfarin therapy. In this study, we investigated 47 patients with effective long-term therapy by warfarin well-controlled by monitoring of International Normalised Ratio (INR). All patients were tested for gene polymorphisms VKORC1, CYP2C9*C2, and CYP2C9*C3, which were used for a dose calculation employing a program www.WarfarinDosing.org. The main goal was to investigate whether the warfarin doses determined by INR are in accordance with the doses calculated according to the pharmacogenetic algorithm. For this purpose, several chemometric tools, namely principal component analysis, cluster analysis, correlation analysis, correspondence analysis, Passing-Bablock regression, Bland-Altman method, descriptive statistics, and ANOVA were used. We also analysed the relationship between the dose of warfarin determined by INR and several constitutional and genetic factors. Statistically significant association between clinically optimized warfarin dose and indication for the treatment, age, and warfarin sensitivity determined by VKORC1, CYP2C9 gene polymorphisms were confirmed. Finally, we confirmed a good concordance between the INR determined warfarin doses and pharmacogenetic approach.

## Introduction

Warfarin is a coumarine anticoagulant acting as vitamin K antagonist, interfering with the recycling of vitamin K in the liver leading to reduced production of several clotting factors as well as two coagulation inhibitors protein C and S. Atrial fibrillation (AF) and heart valve disorders represent most frequent indications for life-long thromboprophylaxis with warfarin; warfarin is also effective in the treatment and prevention of venous thromboembolism (VTE). To optimize the therapeutic effect without increasing the risk of bleeding, a close monitoring of prothrombin time, expressed as INR, is required (1-2). Reliability of INR monitoring is an important factor influencing the suitability of warfarin therapy in the individual patient. Dosing of warfarin may be difficult due to its interaction with commonly used medications and food, which may enhance or reduce its anticoagulation effect (3). The response to warfarin is also influenced by genetically determined enzymes involved in its metabolism, such as CYP2C9 (cytochrome P (450) 2C9) and VKORC1 (vitamin K epoxide reductase complex subunit 1). They are related to a wide inter-individual and intra-individual variability in a warfarin dosing (4-7). 

Recent studies suggest that the pharmacogenetics-guided algorithm may accurately determine warfarin dose and thus reduce adverse events (8-9). This will allow a more personalized approach to the control of anticoagulant therapy. However, due to the lack of evidence based on the randomized studies, the use of pharmacogenetics for warfarin dosing is still uncommon in the routine clinical practice (8).

The aim of this study was to evaluate the relationship between the warfarin dose and factors such as age, gender, body mass index, the clinical reason of treatment, and concomitant therapy with statins and genetic polymorphisms. Another goal was to compare warfarin doses determined by INR – “warfarin determined in clinical practice” with doses determined by a pharmacogenetic algorithm – “warfarin genetically determined”.

## Experimental

*Patients*

Patients from the Department of Internal Medicine, Slovak Medical University, and University Hospital between the January 2008 and January 2013 were included in the study. We investigated the patients with an indication for a long-term warfarin therapy (recurrent VTE and atrial fibrillation) monitored by INR. They were categorized into several groups according to 1) Age: a) older patients ≥ 65 years and b) younger patients (< 65 years); 2) Gender: a) males and b) females; 3) Body mass index (BMI): a) normal weight with BMI < 25, b) overweight /obesity with BMI 25-35, and c) morbid obesity (35 and more); 4) Markers of inherited thrombophilia (FV Leiden and/or prothrombin mutation): a) thrombophilia negative and b) thrombophilia positive; 5) Indication for warfarin: a) atrial fibrillation (AF) and b) venous thromboembolism (VTE); and 6) Co-medication with statins: a) patients using statins and b) non-taking statins. The information about inherited thrombophilia were extracted from the patient´s documentation. All patients were investigated for the presence of VKORC1 and CYP2C9 gene polymorphisms. Sensitivity to warfarin (*Sensitivity*) was introduced as a new variable representing the genetically determined response to warfarin. Two categories of *Sensitivity* were assigned: 1) sensitive patients with at least one warfarin sensitive allele and 2) non-sensitive patients with wild-type allele’s presence.

**Table 1:** Genetic polymorphisms in 47 patients on warfarin therapy studied

**Genotype**	**Alleles**	**N**	**(%)**
VKORC1	homozygot (GG)	17	(36.2)
	heterozygot (G**A**)	23	(48.9)
	homozygot (**AA**)	7	(14.9)
CYP2C9*2	wild-type (CC)	33	(70.2)
	heterozygot (C**T**)	13	(27.7)
	homozygot (**TT**)	1	(2.1)
CYP2C9*3	wild-type (AA)	44	(93.6)
	heterozygot (A**C**)	3	(6.4)
	homozygot (**CC**)	0	(0.0)

*Note*: The patients with positive genotypes (alleles of genotypes marked by bold) were included to the first category of *Sensitivity.*

**Table 2:** Patient’s distributions according to categories of elected factors with clinically optimized warfarin doses (*WC*) and results of elected tests

Categorical variable	Categories	N	WC (mg per day) (Mean ± SD)	ANOVA	Mann-Whitney test
(factor)	Non-sensitive	12	7.50 ± 0.53		
*Sensitivity*				< 0.001^a^	< 0.001^a^
	Sensitive	35	4.46 ± 0.29		
	Non-taking	25	5.21 ± 0.43		
*Statins*				0.568	0.683
	Taking	18	5.60 ± 0.56		
	Atrial fibrillation	28	4.55 ± 0.35		
*Indication*				0.009^a^	0.003^a^
	Thromboembolism	19	6.25 ± 0.52		
	Male	28	5.52 ± 0.46		
*Gender*				0.274	0.434
	Female	19	4.81 ± 0.40		
	Less than 65y	21	6.05 ± 0.51		
*AGE*				0.019^a^	0.024^a^
	65y and more	26	4.57 ± 0.36		
	Normal weight	11	5.47 ± 0.57		

*Note*: SD – standard deviation.

a Statistically significant values *p *< 0.05.

* Result of Kruskal-Wallis test.

**Table 3:** Results of Passing-Bablock method for comparison of *WC *and *WG *in different software’s packages

**Software**	**Intercept A**	**95% CI (A)**	**Slope B**	**95% CI (B)**
*MedCalc*	0.792	(-0.350 to 1.721)	0.828	(0.632 to 1.050)
*Analyse-It*	0.792	(-0.171 to 1.761)	0.828	(0.623 to 1.006)
*XL-STAT*	0.988	(-0.128 to 1.781)	0.800	(0.608 to 1.006)

**Figure 1:**
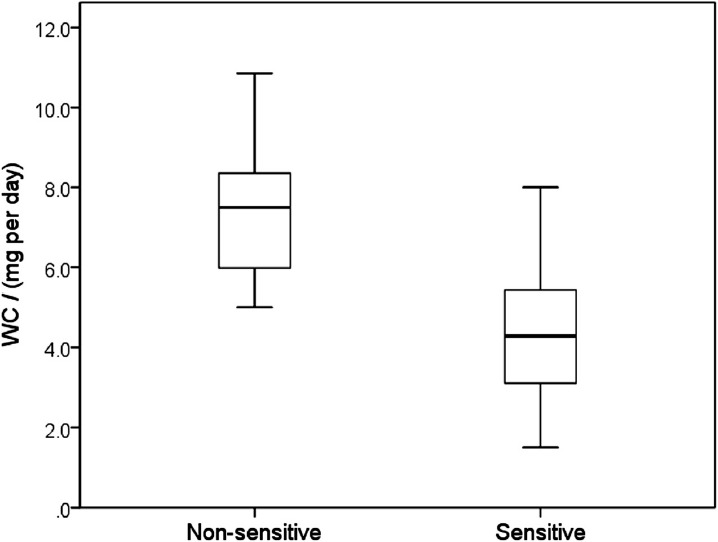
Clinically optimized warfarin dose (*WC*) according to *Sensitivity, *characterized by a presence or absence of warfarin sensitive gene polymorphisms (median, 25 – 75 percentiles, minimum and maximum values)*. *Maximum length of each whisker is 1.5-fold the interquartile range. Sensitive patients required significantly lower warfarin doses presented by fully separated box-plots (*p *< 0.001)

**Figure 2:**
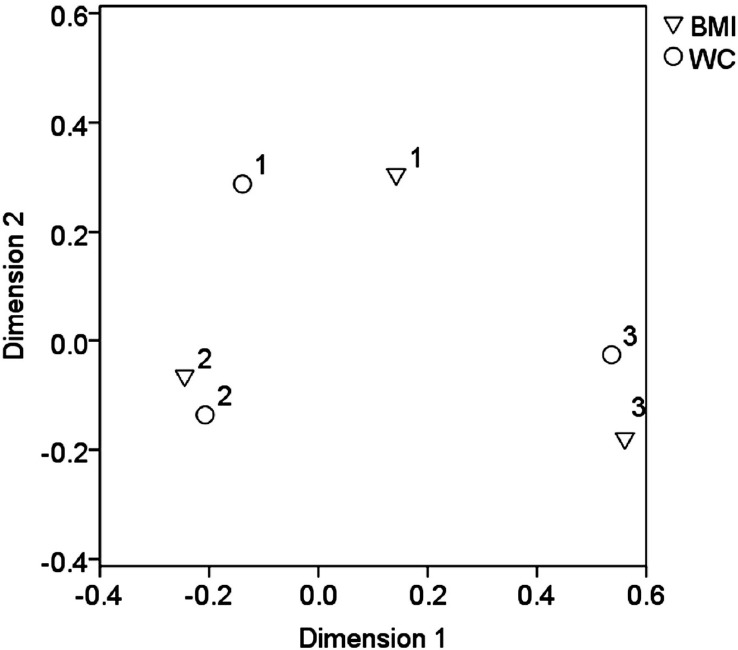
Output of correspondence analysis showing categorical variables *BMI *and *WC *in projections on the first 2 dimensions. Cluster 1 represents patients with normal weight and *WC *less than 3.5 mg per day, cluster 2 the patients with overweight and *WC *in the range 3.5 – 7.5 mg per day and cluster 3 the obesity/morbid obesity and *WC *equal 7.5 mg per day and more

**Figure 3:**
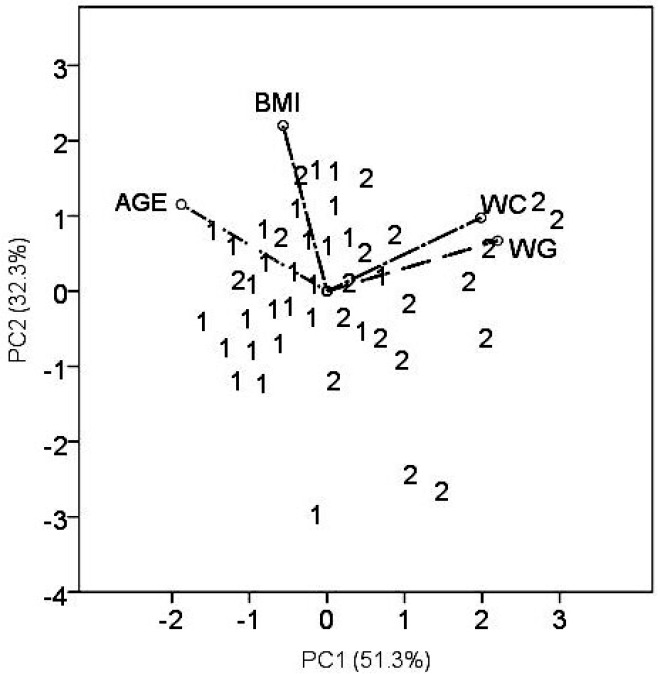
PCA biplot displaying the position of the patient’s samples as well as 4 descriptors in the plane *PC2 *vs. *PC1*. Patient’s samples are denoted according to indication for the anticoagulant therapy by numbers: 1 – atrial fibrilation and 2 – thromboembolism

**Figure 4:**
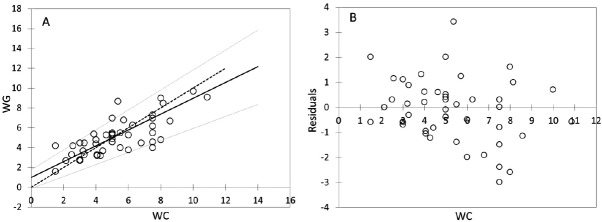
Passing-Bablock regression for comparism *WC *and *WG*, N = 47; (A) Scatter diagram with regression line (dashed line) and confidence bands for regression line. Identity line is solid. Regression line equation: *y *= 0.988 + 0.800*x*; 95% CI for intercept -0.128 to 1.781 and for slope 0.608 to 1.006 indicated good agreement. (B) Residual plot presents distribution of difference around fitted regression line

**Figure 5:**
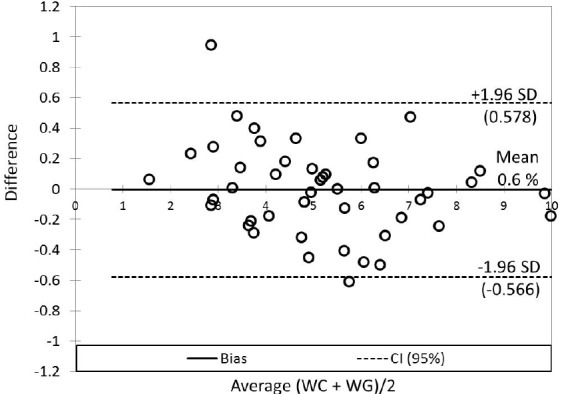
Bland-Altman plot for comparison of the relative differences between *WC *and *WG*. The 95% limits of agreement (SD, dashed lines) contain 95.7% of the difference scores (45 objects from 47). The mean difference (bias, solid line) between *WC *and *WG *was 0.006 (0.6%; 0.166 mg per day)

“*WC*” designates warfarin dose established by “clinical” follow up and INR monitoring. The dose was continued/increased/reduced according to actual INR in order to maintain a therapeutic range between 2.0 and 3.0. In patients using non-constant daily doses of warfarin during a week, the mean daily dose of warfarin (mg per day) was calculated from a total dose per week.

“*WG*” means a dose of warfarin determined by pharmacogenetic algorithm employing publicly available program www.WarfarinDosing.org (10). A dose is adjusted according to several variables including patient’s age, sex, ethnicity, race, weight, height, concomitant medication (statins, amiodarone, and azoles), liver comorbidity, and occurrence of polymorphisms, basal INR and a target INR of 2.5.

Thus, *WC* and *WG* were used as continuous variables (descriptors). According to the clinically determined warfarin dose (*WC*), the patients were divided into three categories: 1) ˂ 3.5 mg per day; 2) 3.5 − 7.5 mg per day; and 3) ≥ 7.5 mg per day. Patient’s categories according to the age, BMI, gender, indication for the treatment and concomitant therapy were used as categorical variables (factors). Only patients with a constant therapeutic range of INR between 2.0 and 3.0 were eligible for final statistical evaluation.

*Laboratory testing and genetic investigation of VKORC1 and CYP2C9 polymorphisms*

Prothrombin time (PT) was tested by a standard method using the reagent Thromborel^®^S and the BCS Coagulation Analyzer (Boehringer Coagulation System). The results were expressed in INR, which is prothrombin time ratio raised to the power of the ISI (International Sensitivity Index) value for the tissue factor reagent used in analytical system. PT testing was performed by laboratories Synlab Slovakia, s.r.o. and Medirex, s.r.o.

DNA was extracted and analysed by routine PCR analysis (NucleoSpin Blood, Macherey-Nagel). Genotyping for the CYP2C9*2 and CYP2C9*3 alleles was done by LightMix Kit human CYP 2C9*2 and CYP 2C9*3 (fy Roche) and for VKORC1 by LightMix Kit human VKORC1 C1173T and G-1639A (fy Roche) using LightCycler 2.0 (fy Roche). Genetic testing was performed in laboratories Gendiagnostica s.r.o. Bratislava Center, Slovakia and Medical Genetics, University Hospital Bratislava, Slovakia.

*Statistical analysis*

For normality’s testing, two methods were performed: relying on statistical tests (Shapiro-Wilk’s test) (11) and visual inspection (Q-Q plots) (12). The impact of the target categorical variable Sensitivity, as well as other factors, was investigated by Analysis of variance (ANOVA) and the non-parametric Kruskal-Wallis test and Mann-Whitney test (for pair-wise comparisons) (13). 

The correspondence analysis was applied for the visualization of the relationship between BMI and *WC*; correlation analysis for evaluation interrelations between *WC* and *WG* was used; principal component analysis for data visualization was applied (14). The comparison of *WC* and *WG* was performed by non-parametric Passing-Bablok method (15). Breusch-Pagan or White tests were used for examination of heteroscedasticity (16). Cusum test was used for the determination of linearity between *WC* and *WG*. Bland-Altman plot (17) was used for visualization differences between *WC* and *WG*, respectively bias determination. A *p*-value of less than 0.05 was considered to be statistically significant. Several commercial software packages were applied, particularly SPSS ver. 19 (18), Analyse-It, ver. 2.12 (19), MedCalc ver. 10.4.0.0 (20), XLSTAT 14.4 (21) and StataIC 12 (22).

## Results

A total number of 47 consecutive patients (28 males and 19 females) with a median age of 68 years (range 25–84 years) were evaluated. In the entire group of patients a stable therapeutic range of INR between 2.0 to 3.0 adjusted according to a regular control of INR (*WC*) was maintained by a daily dose of warfarin of 5.23 ± 2.18 mg. The mean time for a stable dose adjustment was 7.2 ± 5.0 months. However, the doses of *WC* varied widely (range 2.0 to 11.0 mg): 20 patients (42.6%) used a dose < 5.0 mg per day and 27 patients (57.5%) a dose > 5 mg per day. In our data set, a slightly higher proportion of patients (55.3%) was in age over 65 years and with atrial fibrillation (59.6%) as an indication for the treatment. Less than half of all patients (46.8%) were using statin therapy (atorvastatin, fluvastatin, simvastatin) and 2 patients (4.25%) received amiodarone. Most of the patients (48.9%) had sensitive allele A of the VKORC1 gene in a heterozygous combination (G/A) and only 7 patients were homozygous for AA combination. In contrast to VKORC1, most of the patients (70.3% and 93.6%) were homozygous for a wild-type of CYP2C9*2 (CC) and CYP2C9*3 (AA) genes, respectively. A minority of the patients were heterozygous for both CYP2C9 gene polymorphism, with only one homozygote CYP2C9*2 with TT alleles (Table 1).

*Analysis of factors influencing the warfarin dosing*

The women required lower *WC* doses (4.81 ± 0.41 mg per day) than men (5.52 ± 0.46 mg per day) (*p* = 0.274). In patients ≥ 65 years the *WC* was 4.57 ± 0.36 mg per day in contrast to the younger patients with *WC* of 6.05 ± 0.51 mg per day (*p* = 0.019). The maintenance dose of warfarin was also significantly different by in patients classified as “sensitive”, i.e. carrying at least one gene polymorphism increasing the response to warfarin and non-sensitive patients: 4.46 ± 0.29 mg per day versus 7.50 ± 0.53 mg per day (Figure 1, *p* < 0.001) (Table 2).

Statistical analysis did not confirm a significant difference in warfarin dosing between the patients taking and non-taking statins (*p* = 0.568), as well as, a non-significant impact of thrombophilia markers (*p* = 0.781) and BMI (*p* = 0.821) on the warfarin dosing in the *WC *(Table 2). The correspondence analysis showed that the patients with overweight (BMI range 25 – 35) were predominantly using a dose of in the range of 3.5 – 7.5 mg per day (Figure 2). 

The principal component analysis is a multidimensional technique and offers a different views of the data (Figure 3). Simultaneously, the patient samples and variables (*WC,* *WG*, *AGE*, and *BMI*), are presented in the plane of principal components (*PC*). The group of patients with thromboembolism (2) is clustered close to higher warfarin doses (positive values of *PC1*) representing by rays *WC* and *WG*. Patients with atrial fibrillation (1) are grouped close to the ray *AGE* (negative *PC1* values). It indicates that elderly patients required lower warfarin doses, in contrast to younger patients suffering predominantly from VTE.

*Evaluation of agreement warfarin dosing methods*

The mean of *WG* dose was slightly lower (5.07 ± 1.85 mg per day) in contrast to the mean of *WC* dose (5.23 ± 2.18 mg per day). Correlation analysis provided statistically significant mutual correlations between *WC* and *WG* (by non-parametric Spearman correlation analysis: R = 0.743; *p* < 0.001; R_crit_ = 0.294). The Passing - Bablock method indicated relatively good agreement between *WC* and *WG* (Table 3), but the scatter diagram of the method shows the identity line is more diverted at higher warfarin doses (Figure 4A); residual plot (Figure 4B) presents residuals from the fitted regression line, precision all over the measurement range and visually identifies non-constant standard deviation between *WC* and *WG*. The bias (mean difference) in Bland-Altman plot (Figure 5) is shifted slightly from zero (0.006; CI Bias 95% -0.091 to 0.078; 0.6%) and presents the difference -0.166 mg per day *WG* dose from *WC* dose.

## Discussion

Atrial fibrillation (AF) and heart valve disorders represent most frequent indications for life-long thromboprophylaxis with warfarin. The prevalence of AF markedly increases with an older age: about 5% of people over 65 years and 10% of people of age 80 years suffer from AF (23). Venous thromboembolism (VTE), which includes deep vein thrombosis and/or pulmonary embolism, is the third leading cause of cardiovascular morbidity and mortality. Inherited thrombophilia, defined as a genetically determined tendency to develop VTE, contributes to the pathogenesis of 40% of VTE episodes (24-25).

The literature reports, the maintenance dose of warfarin inversely proportional to age (26-28) and women require lower warfarin doses than men (28). Some studies also observed an association between BMI and warfarin dose (28-30). Regarding drug interactions, fluvastatin increases the risk of bleeding due to the strong affinity for the cytochrome P450 enzyme 2D6, while this interaction has not been seen with atorvastatin or simvastatin (31). The genetic polymorphisms also showed an important impact on warfarin dosing. Patients with these polymorphisms require lower doses and are at a greater risk of bleeding during warfarin therapy (4-7).

In the present study, patients with atrial fibrillation required significantly lower warfarin doses (*p* = 0.009), which was also probably influenced by the age factor as this patient group was mostly represented by elderly patients (26-27). We also confirmed the maintenance dose of warfarin is inversely proportional to age (*p* = 0.019) (26-27; 32-33). Statistical analysis did not confirm a significant impact of sex, BMI, and thrombophilia markers on the clinically optimized warfarin dose. Nevertheless, this proportion is higher than in the healthy population, where these markers occur in 6% and 2–3%, respectively. No statistically significant difference in warfarin dosing adjusted by INR was also found between the patients taking and non-taking statins in our cohort (*p* = 0.568), probably due to the heterogeneity of statins used. Regarding the impact of genetic polymorphisms on warfarin dosing (VKORC1, CYP2C9*C2, and CYP2C9*C3), patients with positive at least one allele indicating higher sensitivity to warfarin needed to reduce warfarin doses because increasing the anticoagulant effect of warfarin; “sensitive” patients to warfarin therapy needed significantly lower maintenance warfarin doses (*p* < 0.001), respectively.

In the second part of the study, we investigated the concordance between warfarin doses as determined by a routine clinical practice employing the INR measurements and the pharmacogenetic algorithm. The pharmacogenetics-guided algorithm may accurately determine warfarin dose and reduce adverse events (8-9). Many studies focused on the establishment of an adequate model for determination of warfarin doses using genetic polymorphisms (34-35). The nature of a population plays an important role in choosing the most appropriate pharmacogenetic algorithm, therefore several studies compared the involved pharmacogenetic algorithms (34). One of the most reliable dosing method for predicting warfarin doses within the ideal dose in patients requiring lower warfarin doses (< 3 mg per day) is the Gage et al. algorithm (35-36). The algorithm is similar to the algorithm available at www.WarfarinDosing.org (10). The online version has been expanded to also accommodate newer single-nucleotide polymorphisms that have minor effects on the dose (36).

In this study, the results of several statistical approaches confirmed relatively good agreement between the methods. Therefore, our results support a wider use of pharmacogenetics algorithms for the installation of the initial dose of warfarin in common practice, but it is precluded by the fact that the gene polymorphism investigation and obtaining the results from a central laboratory may take several days and even 1 week. Nonetheless, the pharmacogenetic algorithm may be successfully used for precise determination of maintenance doses of warfarin in patients on long-lasting warfarin therapy. 

The main limitation of the study was a relatively small sample of patients (especially the group of patients with VTE) and retrospective nature of the study.

## Conclusions

We evaluated the relationship between warfarin doses clinically determined according to INR and age, gender, body mass index, indication for the warfarin therapy, statins use, inherited thrombophilia and VKORC1, CYP2C9*C2 and CYP2C9*C3 gene polymorphisms. We also investigated the concordance between warfarin doses as determined by a routine clinical practice employing the INR measurements and the pharmacogenetic algorithm. 

Statistically significant association between clinically optimized warfarin dose and age of patients, indication for the treatment and warfarin sensitivity determined by VKORC1, and CYP2C9 gene polymorphism occurrence was confirmed. We confirmed relatively good concordance between the two methods of warfarin dosing determination. The pharmacogenetic algorithms were predicted in 0.166 mg (0.6%) lower warfarin doses in contrast to the clinical approach. These results suggest that the pharmacogenetic algorithm will offer similar warfarin doses as determined by a routine clinical practice employing the INR measurements. In patients with higher warfarin doses required, we suggest employing other investigations realized on bigger dataset, e.g. Passing-Bablok method, by which bigger differences are visible.
